# Novel lateral flow assay for point-of-care detection of *Neisseria gonorrhoeae* infection in syndromic management settings: a cross-sectional performance evaluation

**DOI:** 10.1016/S0140-6736(23)02240-7

**Published:** 2024-02-06

**Authors:** Remco P H Peters, Jeffrey D Klausner, Laura Mazzola, Mandisa M Mdingi, Hyunsul Jung, Ranjana M S Gigi, Jeremie Piton, Joseph Daniels, Lindsey de Vos, Paul C Adamson, Birgitta Gleeson, Cecilia Ferreyra

**Affiliations:** Foundation for Professional Development, Research Unit, East London, South Africa (Prof R P H Peters PhD, M M Mdingi MSc, R M S Gigi MMed, L de Vos MSc); Department of Medical Microbiology, University of Pretoria, Pretoria, South Africa (Prof R P H Peters, H Jung PhD); Division of Medical Microbiology, University of Cape Town, Cape Town, South Africa (Prof R P H Peters); Keck School of Medicine, Department of Population and Public Health Sciences, University of Southern California, Los Angeles, CA, USA (Prof J D Klausner MD); Foundation for Innovative New Diagnostics (FIND), Geneva, Switzerland (L Mazzola PhD, J Piton PhD, B Gleeson PhD, C Ferreyra MD); Department of Social and Preventive Medicine, University of Bern, Bern, Switzerland (R M S Gigi); Edson College of Nursing and Health Innovation, Arizona State University, Phoenix, AZ, USA (J Daniels PhD); David Geffen School of Medicine, University of California, Los Angeles, CA, USA (P C Adamson MD)

## Abstract

**Background:**

A rapid and affordable point-of-care test is a priority for *Neisseria gonorrhoeae* control. WHO and Foundation for Innovative New Diagnostics (FIND) have a target product profile for a non-molecular *N gonorrhoeae* rapid point-of-care test that requires a clinical sensitivity of greater than 80% and a specificity over 95% to be considered useful in syndromic management; test turnaround time should be 30 min or under, and the test should cost less than US$3. A novel lateral flow assay (LFA) was developed to achieve that profile.

**Methods:**

In this cross-sectional study we evaluated the performance of the novel *N gonorrhoeae* lateral flow assay (NG-LFA) at the primary health-care level in South Africa. Male patients with urethral discharge syndrome and female patients with vaginal discharge syndrome were recruited from five primary health-care facilities in the Buffalo City Metropolitan Municipality health district of South Africa. First-void urine specimens and nurse-collected vaginal swabs were tested in-facility with the NG-LFA and Xpert CT/NG PCR assay. *N gonorrhoeae* multi-antigen sequence typing (NG-MAST) was performed on all LFA positive specimens.

**Findings:**

Between March 7, and Sept 19, 2022, we enrolled 200 male patients with urethral discharge and 200 female patients with vaginal discharge. The median age of male patients was 24 years (IQR 21–31 years), and the median age of female patients was 25 years (IQR 21–32 years). In addition, 23 male patients and 12 female patients who presented at the facility with a partner notification slip were enrolled of whom one (4%) and five (42%) were symptomatic, respectively. NG-LFA and Xpert results were available for all participants. In urine specimens, NG-LFA sensitivity was 96·1% (Wilson 95% CI 91·2–98·3; 123 LFA-positive among 128 PCR-positive specimens) and 91·7% in vaginal swab specimens (78·2–97·1; 33 LFA-positive among 36 PCR-positive). The specificity was 97·2% in urine specimens (90·4–99·2; 70 LFA-negative among 72 PCR-negative) and 96·3% in vaginal specimens (92·2–98·3; 158 LFA-negative among 164 PCR-negative). In 156 LFA-positive specimens, NG-MAST showed 93 different sequence types.

**Interpretation:**

The novel NG-LFA had excellent clinical sensitivity and specificity in symptomatic male and female patients. The test met the optimal requirement for sensitivity and the minimal requirement for specificity specified in the target product profile. NG-LFA could provide an important tool to optimise clinical management and reduce excess antibiotic use in settings without direct access to laboratory testing.

**Funding:**

Global Antimicrobial Resistance Innovation Fund (GAMRIF) via FIND and National Institutes of Health.

## Introduction

WHO estimates that 87 million new cases of gonorrhoea occur worldwide annually, with low-income and middle-income countries disproportionally affected.^1^ In South Africa, less than 30% of the *Neisseria gonorrhoeae* cases are reported as treated.^2^ Untreated *N gonorrhoeae* infections are associated with epididymitis, pelvic inflammatory disease, ectopic pregnancy, infertility, and adverse pregnancy outcomes, and infection enhances the transmission and acquisition of HIV.^3,4^ Additionally, the increasing emergence and spread of antimicrobial resistance of *N gonorrhoeae* to most available antibiotics is a global public health concern.^5^

The syndromic approach has been recommended by WHO since the 1990s to treat individuals with symptoms and signs suggestive of a sexually transmitted infection (STI) in low-income and middle-income countries. Without access to affordable diagnostic tests, male patients presenting with urethral discharge syndrome and female patients with vaginal discharge syndrome are treated with an empirical combination of antibiotics that covers the most likely aetiology.^6^ The syndromic management algorithm can differ between countries, and over time based on the local epidemiology and available resources.^7,8^ However, while sensitive for the management of *N gonorrhoeae*-associated urogenital discharge, the syndromic approach could result in the excessive use of antibiotics and drive antimicrobial resistance.

Molecular tests are widely used to diagnose STIs in settings where resources are available, but this is not feasible in most lower-income settings due to the cost and lack of laboratory access. The development and implementation of a low-cost, rapid STI point-of-care test as an alternative to molecular tests is a priority to optimise syndromic management strategies and to improve health outcomes. The Foundation for Innovative New Diagnostics (FIND), together with WHO, developed a target product profile for *N gonorrhoeae* diagnostics at the primary health-care level. The minimum requirement of a non-molecular rapid point-of-care STI test to be considered useful for syndromic management is a turnaround time of 30 min or faster, with test sensitivity greater than 80% and specificity greater than 95%; the optimum requirement for the test would be 10 min or faster from specimen collection to result, with test sensitivity 90% or greater, and specificity 98% or greater.^9^ Ideally, the test would not require any additional equipment, but use of a reusable electronic reader is considered appropriate if it improves test performance.^9^

Lateral flow assays (LFAs) and other immunoassays are non-molecular antigen detection tests that can be used for low-cost identification of *N gonorrhoeae*. Several immuno-assays for *N gonorrhoeae* have been evaluated in the past; these showed good specificity, but low sensitivity.^10,11^ To address the diagnostic gap, FIND led the development of a novel *N gonorrhoeae* lateral flow assay (NG-LFA) in line with the target profile for point-of-care STI testing in syndromic management settings.^9^ The NG-LFA demonstrated promising performance for frozen urine clinical specimens (n=40), where the NG-LFA was positive in 39 (97·5.%) of 40 samples and negative in 40 (100%) of 40 samples tested.^12^

Based on the promising results of the laboratory evaluation of the LFA, we conducted a clinical study to determine the diagnostic performance of the NG-LFA for *N gonorrhoeae* in male patients presenting with urethral discharge and female patients with vaginal discharge at the primary health-care level in South Africa.

## Methods

### Study design and participants

We conducted a cross-sectional evaluation study at five primary health-care facilities in the Buffalo City Metropolitan Municipality health district of South Africa. The study was approved by the Human Research Ethics Committee of the Faculty of Health Sciences at the University of Pretoria (510/2021) and by the local government health authorities.

Patients presenting to participating centres were confirmed for urethral discharge (for male patients) or vaginal discharge (for female patients) by the research nurse on physical evaluation. If the patient was an adult (aged 18 years or older) and presenting with urethral or vaginal discharge they were eligible to participate in the study. In addition, we recruited symptomatic and asymptomatic individuals visiting the facility with a partner notification slip; these slips are provided to patients with genital discharge to give to their sexual partners to prompt them to seek treatment as part of routine care. Individuals were only allowed to participate once. Although we did not record the number of individuals presenting with genital discharge at participating facilities during the study period, we estimate that over 80% were enrolled; study uptake was 100% among those presenting to the research team. Written informed consent was obtained from all study participants.

### Procedures

The NG-LFA was developed by FIND in collaboration with DCN Diagnostics (Carlsbad, CA, USA) to detect an *N gonorrhoeae* surface-exposed antigen in specimens from urine and vaginal swabs. To improve the sensitivity of the test, the NG-LFA uses a europium-based conjugate. Laboratory evaluation showed a limit of detection of 1–5 × 10^3^ colony forming units per mL.^12^ Preliminary assay specificity was 100% for non-*Neisseria* bacteria, but cross-reaction was observed with *Neisseria meningitidis*, *Neisseria lactamica*, and *Neisseria polysacchareae*;^12^ although urogenital infection with these bacteria is rare.

The NG-LFA uses a sample tube in which 0·5 mL urine is mixed with 0·8 mL buffer; vaginal swabs are eluted in 0·8 mL buffer (figure). Using a dropper, the test cassette is inoculated with four drops of fluid from the sample tube. After 20 min, the cassette is inserted in a handheld electronic fluorescence reader that provides an automated qualitative result (positive, negative, or invalid) for *N gonorrhoeae* detection (appendix p 1). The handheld reader is an essential test component as fluorescence is invisible to the eye; it is powered by an external battery (requiring charge from the electricity network once every 3–4 weeks during our study), and it does not require internet connectivity. It reads one test at a time, but only takes a few seconds per cassette. The device records fluorescence data for each control-line and test-line on the cassette which can be downloaded from the reader. Test results do not require any operator interpretation; the qualitative results were manually recorded by the study team. At enrolment, a short questionnaire of demographic characteristics and clinical presentation was completed by the research team. Trained study nurses and fieldworkers performed LFA and Xpert testing at the facility.

Male patients were asked to provide a first-void urine specimen, which was subsequently split into three aliquots. Nurses collected three vaginal swabs from each female participant. One urine aliquot or a rayon swab was prepared for immediate NG-LFA testing. A second specimen was processed using the Xpert urine collection or endocervical specimen collection kit (Cepheid, Sunnyvale, USA) and tested on-site by trained staff with the Xpert CT/NG PCR assay on a Gene Xpert IV machine (Cepheid, Sunnyvale, USA) per the manufacturer’s instructions. The Xpert CT/NG assay provides an automated result (positive, negative, invalid, or error) and does not require the user to interpret data. This automated result was recorded by the study team. The test was repeated in case of an invalid result.

The third urine aliquot and a flocked vaginal swab (Copan, Brescia, Italy) were stored at 4°C and shipped within seven days of collection to the University of Pretoria for further microbiological evaluation. An additional urethral specimen (from male patients) or vaginal specimen (from female patients) was obtained on the same day for *N gonorrhoeae* culture in the case of a discordant result between the NG-LFA and Xpert *N gonorrhoeae* result; these were directly inoculated on New York City medium agar and transported using a CO_2_ container to the PathCare laboratory in East London, South Africa, for incubation and culture.

While the NG-LFA results were not used for clinical management, all participants with a positive Xpert PCR result were provided with pathogen-directed therapy;^6^ participants who were not willing to wait for their result were provided with syndromic treatment as per South African guidelines.^6^

In the case of a discordant result between the NG-LFA and Xpert we performed the following tests: Lightmix Kit 480 HT CT and NG assay (TIB MOLBIOL, Berlin, Germany) on the LightCycler (Roche Diagnostics, Risch-Rotkreuz, Switzerland) to confirm presence of *N gonorrhoeae*, and PCR for detection of *Neisseria* species, *N meningitidis*, and *N polysacchareae*.^13–15^

Culture-free *N gonorrhoeae* multi-antigen sequence typing (NG-MAST), or whole-genome sequencing if the strain isolate was available, was performed to determine sequence types detected by the LFA.^16,17^

### Statistical analysis

We hypothesised that the diagnostic test performance would meet the minimal criteria in the WHO and FIND target product profile. Sample size was based on a 95% CI lower limit of detection of 80% sensitivity as per target product profile, and a minimal specificity of 90%, based on preliminary laboratory evaluations. With the expected test performance of 90% sensitivity and 95% specificity, a sample of 200 symptomatic men (60% estimated prevalence) and 200 symptomatic women (20% estimated prevalence) was considered sufficient. Data were double entered into REDCap (Vanderbilt University, Nashville TN, USA), and checks for consistency and range were performed. Analysis was done using SPSS version 28. Data are presented as number (%) and median (IQR). Comparison of dichotomous variables between groups was done with odds ratios (ORs) with 95% CIs.

Standard 2 × 2 tables were generated to calculate sensitivity, specificity, positive predictive value (PPV), negative predictive value (NPV), and accuracy of NG-LFA compared with the Xpert *N gonorrhoeae* PCR assay result, with 95% Wilson CIs. The Xpert CT/NG assay was used as the gold standard in this evaluation for availability and feasibility reasons. Xpert CT/NG is a United States Food and Drug Administration (FDA)-approved test that can reliably be operated by non-clinical staff.^18^ Studies have reported greater than 98% sensitivity and over 99% specificity for detection of *N gonorrhoeae* in urine and vaginal swab specimens compared with other nucleic acid amplification tests.^19,20^

### Role of the funding source

The funders of the study had no role in study design, data collection, data analysis, data interpretation, or writing of the report.

## Results

Between March 7, and Sept 19, 2022, we enrolled 200 male patients with urethral discharge and 200 female patients with vaginal discharge (table 1). The median age of male patients was 24 years (IQR 21–31 years), 15 (8%) were living with HIV infection, and 58 (29%) reported treatment for urethral discharge in the previous year. The median age of female patients was 25 years (IQR 21–32 years), 34 (17%) were living with HIV infection, and 40 (20%) reported treatment for vaginal discharge in the past year. There were no differences in demographic characteristics between the five primary health-care facilities. We did not observe any adverse events or protocol deviations during this study.

In addition, 23 male patients and 12 female patients who presented at the facility with a partner notification slip were enrolled of whom one (4%) and five (42%) were symptomatic, respectively (separately reported from the main analysis).

NG-LFA and Xpert results were available for all participants; one specimen had an invalid result in Xpert and was negative upon retest. The *N gonorrhoeae* prevalence in male patients was 128 (64%) of 200 by Xpert NG-LFA, and LFA-positive in 123 (96%) of 128 male patients (95% CI 91·2–98·3) with a positive Xpert *N gonorrhoeae* result, and negative in 70 (97%) of 72 male patients (90·4–99·2) with a negative Xpert *N gonorrhoeae* result (table 2). Xpert was positive for *Chlamydia trachomatis* in 47 (24%) of 200 male patients; and 34 (27%) of 128 male patients with *N gonorrhoeae* had concurrent *C trachomatis* infection.

The prevalence of *N gonorrhoeae* in female patients was 36 (18%) of 200 specimens by Xpert NG-LFA positive results, and LFA-positive in 33 (92%) of 36 female patients (95% CI 78·2–97·1) with a positive Xpert *N gonorrhoeae* result, and negative in 158 (96%) of 164 female patients (92·2–98·3) of females with a negative Xpert *N gonorrhoeae* result. Xpert was positive for *C trachomatis* in 35 (18%) of 200 female patients; 17 (47%) of 36 female patients with *N gonorrhoeae* had concurrent *C trachomatis* infection.

The NG-LFA test performance characteristics showed overall excellent sensitivity and specificity in symptomatic patients of both sexes (table 3). NG-LFA test accuracy was 96·5% (95% CI 93·0–98·3) for symptomatic male patients and 95·5% (91·7–97·7) for symptomatic female patients.

The NG-LFA and Xpert *N gonorrhoeae* results were fully concordant for the 35 individuals presenting with a partner notification slip; three (25%) of the 12 female patients had *N gonorrhoeae* detected by both methods, and one (4%) of 23 male patients also had positive results for both methods; these four *N gonorrhoeae* infections were all asymptomatic. The NG-LFA sensitivity and specificity were used to calculate a range of hypothetical PPV and NPV for males and females with genital discharge syndrome (appendix p 2).

Discordant results between NG-LFA and Xpert *N gonorrhoeae* results were observed in 1G participants: seven in male patients and nine in female patients (table 4). In eight cases the NG-LFA was negative with a positive Xpert *N gonorrhoeae* result, while the NG-LFA was positive in another eight cases with a negative Xpert *N gonorrhoeae* result. *N gonorrhoeae* was isolated in three of seven cultures from individuals with a positive Xpert and a negative NG-LFA result; none of the cultures from individuals with positive LFA and negative Xpert grew *N gonorrhoeae*.

Laboratory PCR of the third specimens was positive for *N gonorrhoeae* in nine cases, confirming the positive *N gonorrhoeae* result in three cases of positive NG-LFA and in six cases by Xpert. One participant with a positive Xpert *N gonorrhoeae* result but negative NG-LFA had a positive PCR for *N polysacchareae,* with negative PCR for *N gonorrhoeae* and failed NG-MAST. *N meningitidis* or other *Neisseria* species were not detected in any of the discordant specimens.

Altogether, the results of microbiological investigation suggest that NG-LFA could be classified as true-positive in three discordant cases, false-positive in five, true-negative in one, and false-negative in six of the participants with discordant results between NG-LFA and Xpert. Two participants had a relatively low bacterial load based on their Xpert cycle threshold value.

NG-MAST typing was successful in 156 (93%) of 168 individuals with positive LFA test (164 symptomatic patients and four people presenting with partner notification slip); 93 different NG-MAST types were detected, and one individual had multiple NG-MAST types (appendix pp 3–6).

## Discussion

Affordable and rapid point-of-care *N gonorrhoeae* tests are required to reduce the global burden of infection, reduce *N gonorrhoeae* antimicrobial resistance, and improve health outcomes in settings where syndromic management is standard of care.^3,9^ This study demonstrates that the novel NG-LFA was highly accurate for the rapid detection of *N gonorrhoeae* in symptomatic male and female patients at the primary health-care level in South Africa.

The diagnostic performance of NG-LFA was much better than reported for previous immunoassays for *N gonorrhoeae* (<50% sensitivity compared to nucleic acid amplification tests),^10,11^ and slightly higher than reported for a lateral flow assay for the detection of *N meningitidis* in cerebrospinal fluid of patients with meningitis (95% sensitivity and 90% specificity).^21^ The automated fluorescent reader with a built-in algorithm to classify test results as positive or negative might enhance test sensitivity, especially in specimens with fluorescence measured close to the threshold value.

The observed sensitivity of the NG-LFA meets the target product profile’s optimal requirement (>90%) for use in settings where syndromic management is currently used, while the observed specificity met the minimum requirement (>95%), noting that the lower estimate of the confidence interval for test sensitivity in vaginal swabs was 78·2%. Although the overall performance was excellent, the observed sensitivity and specificity were slightly higher in urine samples than vaginal swabs; this is possibly due to the nature of vaginal swab specimens, which have many cell types, vaginal extracellular matrix, and other bacteria present that could interfere with the immunoassay binding process. NG-LFA and Xpert *N gonorrhoeae* results were automatically generated by each device, without operator involvement, and were available for all participants. This makes verification and interpretation bias unlikely, despite lack of masking.

We observed several discordant results between NG-LFA and Xpert *N gonorrhoeae* testing. Microbiological analysis of the third stored specimen at the University of Pretoria showed that NG-LFA could be interpreted as true-positive in three out of 1G cases. Four false-positive NG-LFA results were observed for vaginal swabs but cross-reaction with other *Neisseria* species could not be identified; this might be caused by unknown interfering substances, atypical binding, or unknown cross-reaction due to the nature of these specimens. One probable false-positive Xpert result was observed for a possible case of *N polysacchareae* infection. False-negative LFA results might have been due to low-load specimens or the absence of NG target gene expression. A large diversity of *N gonorrhoeae* sequence types was detected by LFA suggesting broad local strain coverage.

This study had several limitations. First, laboratory-based *N gonorrhoeae* PCR was only performed on discordant specimens, and we did not include a separate reference test. This did not allow comparison of test performance between the NG-LFA and Xpert assay, although Xpert is the FDA-approved reference standard test that can be considered a gold standard. Second, we did not perform *Neisseria* species culture or in-depth microbiological analysis of *Neisseria* species in discordant specimens. It cannot be ruled out that cross-reaction due to non-gonococcal *Neisseria* species was missed in some discordant cases. We also used vaginal swabs, not cervical swab specimens for culture of *N gonorrhoeae* from women with discordant results. Although cervical swabs provide optimal results, vaginal swabs are more convenient, and have a lower chance of discouraging participants, as speculum investigation has a negative reputation in our health-care setting. We used health-care provider collected swabs to ascertain the quality of all three vaginal specimens. Self-collected vaginal swabs could be more feasible during potential future implementation,^22^ and we do not anticipate diagnostic performance to be different if swabs are self-collected. Male self-collected meatal swabs could also be considered an alternative to urine samples. Third, this evaluation was conducted in one region of South Africa; studies from other settings are required to replicate test performance. Finally, although not a study limitation, a test that concurrently detects *C trachomatis* is preferential from an implementation perspective due to the high burden of infection and co-infection with *N gonorrhoeae*.

Research studies using in-facility Xpert assay testing have shown major benefits of STI point-of-care diagnostic testing on the time to treatment in syndromic management settings.^23–26^ Another recent development is the Visby Medical Sexual Health Test; a rapid single-use molecular point-of-care test for multiple STIs.^27^ Despite high test performance of these assays, molecular test technology is likely to remain too expensive for widespread use in lower-income settings. Therefore, implementation of a non-molecular test should be considered to optimise STI diagnosis and treatment with the further benefit of reducing excess antibiotic use, thereby promoting antimicrobial stewardship in syndromic management settings.^28^

The NG-LFA meets most of WHO’s ASSURED criteria for the ideal rapid point-of-care test in lower income countries.^29^ The test sensitivity and specificity were excellent, the test was considered easy to use,^30^ and it was implemented at the primary health-care level by the appropriate staff cadres. Test results were available within 30 min, in line with the minimum test requirement. The ideal rapid point-of-care STI test would not require additional equipment, but the NG-LFA reader is considered essential for test performance. From an affordability perspective, the NG-LFA was developed to cost less than US$3 per test. The cost of the NG-LFA would probably need to be further reduced; other lateral flow tests are considered affordable at $0·50–1·00 for widespread use to aid in the diagnosis of infection with HIV, hepatitis B, hepatitis C, and malaria. ^29^ Price reduction will be directly related to high volume purchasing, hence the need to include such testing in global guidelines and other multi-disease global programmes.

The NG-LFA provides a major step forward in optimising syndromic management and antimicrobial stewardship in lower-middle and low-income countries. In symptomatic males, the NG-LFA could be used to guide antimicrobial treatment decisions, especially in relation to the provision of ceftriaxone or use of new antibiotics in the pipeline. Given the high NPV of a negative NG-LFA result, ceftriaxone could be omitted in the treatment of women with vaginal discharge and a negative test result. This approach, using an affordable, accurate non-molecular test to optimise syndromic management, has been incorporated as option two in the most recent WHO guidelines for management of vaginal discharge.^31^ Other potential uses of NG-LFA could be to optimise antimicrobial surveillance systems (eg, collect a specimen for antimicrobial culture only in individuals with a positive NG-LFA result) and to facilitate recruitment of individuals in *N gonorrhoeae* treatment trials. A study-evaluating diagnostic performance of NG-LFA for testing asymptomatic individuals has recently commenced.

In conclusion, the NG-LFA is the first non-molecular point-of-care test that has excellent diagnostic accuracy for the detection of *N gonorrhoeae,* and it provides a major step forward in moving from syndromic to aetiological management of STIs in lower-income settings.

## Supplementary Material

Appendix

## Figures and Tables

**Figure:**
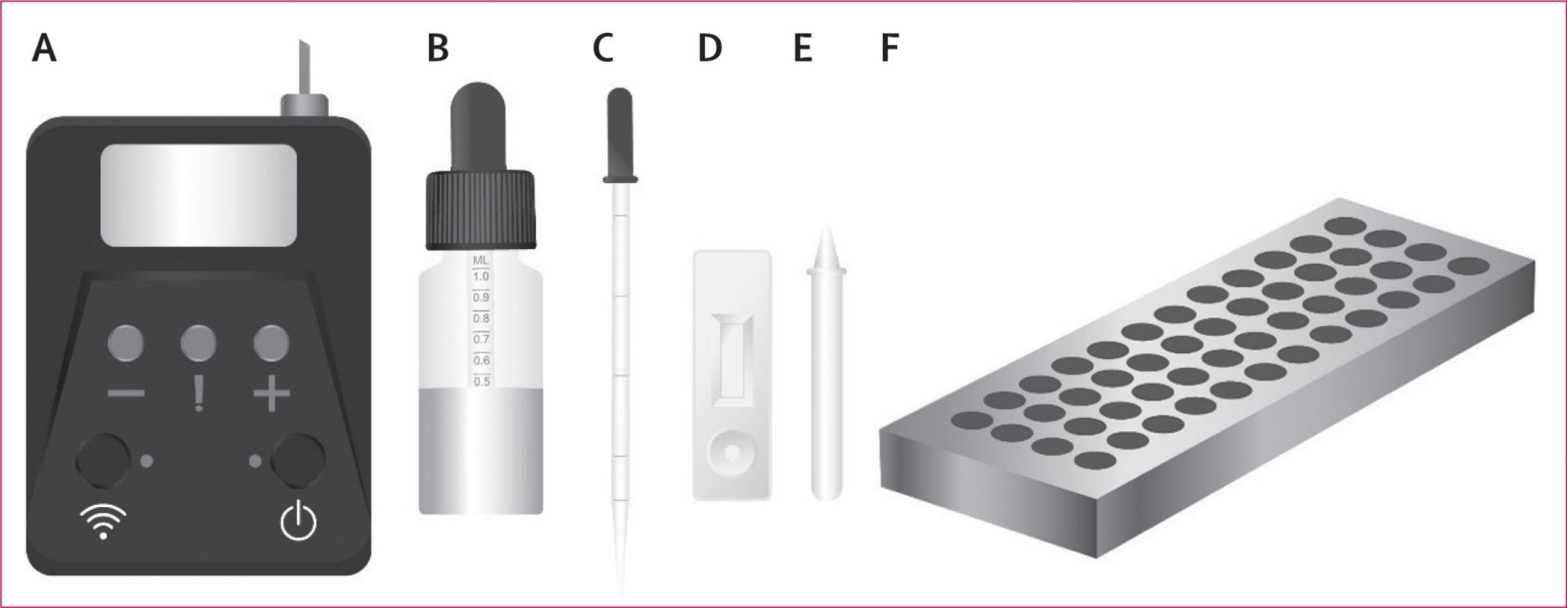
*Neisseria gonorrhoeae* lateral flow assay testing device and resources (A) Automated reader, (B) buffer bottle, (C) pipette, (D) test cassette, (E) dropper, (F) specimen block.

**Table 1:** Characteristics of the study population in South Africa

	Male patients with urethral discharge (n=200)	Female patients with vaginal discharge (n=200)
Age, years	24 (21–31)	25 (21–32)
Employment status		
Formal/self-employment	90 (45%)	59 (30%)
Unemployed	79 (40%)	76 (38%)
Student	31 (16%)	65 (33%)
Relationship status		
Single	68 (34%)	55 (28%)
Steady partner	107 (54%)	105 (53%)
Married or living together	25 (13%)	40 (20%)
Sex partners		
Steady partner only	73 (37%)	133 (67%)
Occasional partners	55 (28%)	45 (23%)
Steady and occasional partners	72 (36%)	22 (11%)
Condom use last sex act		
Yes	41 (21%)	30 (15%)
No	159 (80%)	170 (85%)
HIV-infection status		
HIV-positive; on treatment	11 (6%)	29 (15%)
HIV-positive; not on treatment	4 (2%)	5 (3%)
HIV-negative	172 (86%)	155 (78%)
Refused to disclose or test	13 (7%)	11 (6%)
History of treatment for genitourinary discharge		
Treated past 30 days	11 (6%)	10 (5%)
Treated past 1–12 months	47 (24%)	30 (15%)
Treated more than a year ago	34 (17%)	39 (20%)
Never previously treated	108 (54%)	121 (61%)

Data are n (%) or median (IQR).

**Table 2:** Performance of the lateral flow assay compared to Xpert for detection of *Neisseria gonorrhoeae*

	Xpert positive	Xpert negative	Total
**In urine specimens from male patients with urethral discharge (n=200)**			
LFA positive	123	2	125
LFA negative	5	70	75
Total	128	72	200
**In vaginal swabs from female patients with vaginal discharge (n=200)**			
LFA positive	33	6	39
LFA negative	3	158	161
Total	36	164	200

LFA=lateral flow assay.

**Table 3:** Diagnostic performance characteristics of *Neisseria gonorrhoeae* lateral flow assay compared with Xpert for the detection of *N gonorrhoeae* in symptomatic male and female patients

	Male patients (n=200)	Female patients (n=200)
*Neisseria gonorrhoeae* prevalence	128 (64%)	36 (18%)
Sensitivity	96·1% (91·2– 98·3)	91·7% (78·2–97·1)
Specificity	97·2% (90·4–99·2)	96·3% (92·2–98·3)
Positive predictive value	98·4% (94·4–99·6)	84·6% (70·3–92·8)
Negative predictive value	93·3% (85·3– 97·1)	98·1% (94·7–99·4)
Accuracy	96·5% (93·0–98·3)	95·5% (91·7–97·7)

Data n (%) or % (95% Wilson’s CI).

**Table 4:** Microbiological analysis of specimens with discordant results between NG LFA and Xpert for *Neisseria gonorrhoeae*

	LFA result	LFA fluorescent intensity value*	Xpert result	Ct-value (NG2/NG4)	NG PCR	*Neisseria* species PCRs	NG-MAST ST	Comment
**In urine samples from males with urethral discharge (n=7)**								
Participant 1	Negative	0·06271	Positive	34·4/34·7	Positive	Not detected	21260	False-negative LFA
Participant 2	Negative	0·00029	Positive	30·3/29·9	Positive	Not detected	21276	False-negative LFA
Participant 3	Negative	0·00270	Positive	31·0/30·8	Positive	Not detected	Failed	False-negative LFA
Participant 4	Negative	−0·02429	Positive	21·1/20·6	Positive	Not detected	21255	False-negative LFA
Participant 5	Negative	0·04756	Positive	25·6/25·2	Negative	NS detected	Failed	True-negative LFA
Participant 6	Positive	1·49049	Negative	NA	Positive	Not detected	2975	True-positive LFA
Participant 7	Positive	2·26541	Negative	NA	Negative	Not detected	Failed	False-positive LFA
**In vaginal swab samples from women with vaginal discharge (n=9)**								
Participant 1	Negative	0·00301	Positive	34·9/34·1	Negative	Not detected	Failed	True-negative LFA
Participant 2	Negative	0·01013	Positive	26·9/26·9	Positive	Not detected	18259	False-negative LFA
Participant 3	Negative	0·02899	Positive	28·5/27·8	Positive	Not detected	2187	False-negative LFA
Participant 4	Positive	0·08632	Negative	NA	Negative	Not detected	Failed	False-positive LFA
Participant 5	Positive	0·19150	Negative	NA	Negative	Not detected	Failed	False-positive LFA
Participant 6	Positive	0·11285	Negative	NA	Negative	Not detected	Failed	False-positive LFA
Participant 7	Positive	0·15010	Negative	NA	Negative	Not detected	Failed	False-positive LFA
Participant 8	Positive	0·18251	Negative	NA	Positive	Not detected	Failed	True-positive LFA
Participant 9	Positive	0·07521	Negative	NA	Positive	Not detected	21253	True-positive LFA

LFA=lateral flow assay. Ct=cycle threshold value. NG=*Neisseria gonorrhoeae*. PCR=polymerase chain reaction. NG-MAST=*Neisseria gonorrhoeae* multi-antigen sequence typing. ST=sequency type. NS=*Neisseria polysacchareae*. NA=not applicable.

* Fluorescence threshold for positive reading is ≥0·063.

## Research in context

### Evidence before this study

There is currently no inexpensive, fast, and high sensitivity and specificity non-molecular point-of-care test for the diagnosis of infection with *Neisseria gonorrhoeae,* the lack of which leads to syndromic treatment, increasing overuse of antibiotics and associated resistance. We searched PubMed for publications of lateral flow assay (LFA) and other point-of-care tests for the detection of *N gonorrhoeae* from Jan 1, 2017, to March 1, 2022, with the search terms [“point-of-care” AND “*Neisseria gonorrhoeae*”], and [“lateral flow assay” AND “*Neisseria gonorrhoeae*”] without language restrictions. Our search yielded 106 published articles, of which 27 were not relevant to the topic, and 38 were a study protocol, review or opinion article, or described mathematical modelling results. Of 41 articles with original data, 20 were laboratory test evaluations and 15 addressed implementation issues. We identified six articles describing performance evaluation of a point-of-care test, but all of these were molecular tests; we did not identify any articles describing the evaluation of a lateral flow assay or other non-molecular test for point-of-care detection of *N gonorrhoeae*.

### Added value of this study

We evaluated the novel LFA for point-of-care detection of *N gonorrhoeae* infection in symptomatic men and women in South Africa. Our results show an excellent diagnostic performance for detection of *N gonorrhoeae* in male patients presenting with urethral discharge syndrome and female patients presenting with vaginal discharge syndrome, meeting the optimal requirement for sensitivity and minimal requirement for specificity in the WHO and FIND target product profile. This makes our novel LFA the first point-of-care test for *N gonorrhoeae* globally that meets the profile for use in syndromic management settings.

### Implications of all the available evidence

Based on the excellent diagnostic performance, this novel LFA could be used to optimise STI treatment in lower-income settings without direct access to laboratory testing. This novel LFA provides an opportunity to transform sexually transmitted infection control globally, by accelerating the switch from syndromic to aetiological management and reducing unnecessary use of antibiotics.
