# Supplementary material for: Novel lateral flow assay for point-of-care detection of Neisseria gonorrhoeae infection in syndromic management settings: a cross-sectional performance evaluation
Source: Lancet. Author manuscript; Available in PMC 2024 Jul 14. (PMC11246789; doi:10.1016/S0140-6736(23)02240-7)
Supplement: Appendix [file NIHMS2002671-supplement-Appendix.pdf]

THE LANCET

Supplementary appendix

This appendix formed part of the original submission and has been peer reviewed. We post it as supplied by the authors.

Peters RPH, Klausner JD, Mazzola L, et al. Novel lateral flow assay for point-of-care detection of *Neisseria gonorrhoeae* infection in syndromic management settings: a cross-sectional performance evaluation. *Lancet* 2024; published online Feb 6. https://doi.org/10.1016/S0140-6736(23)02240-7.

ONLINE APPENDIX OF SUPPLEMENTARY MATERIAL

Fig S1……………………………………………2

Table S1………………………………………..3

Table S2………………………………………..4

Fig S1. ***Neisseria gonorrhoeae*** **lateral flow assay automated reader showing a positive and negative result.**

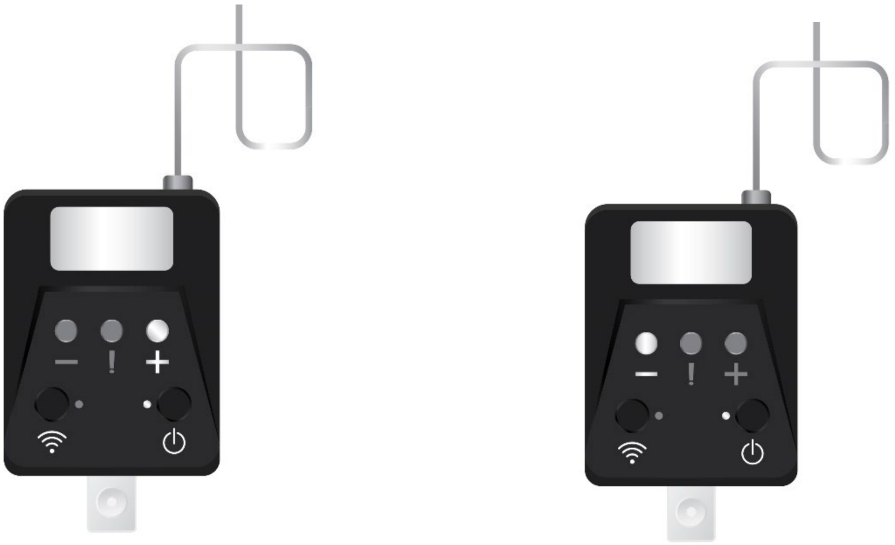

Table S1. Hypothetical estimates of positive and negative predictive values for different prevalence of *Neisseria gonorrhoeae* infection

Prevalence	Males with urethral discharge		Females with vaginal discharge	
	**PPV**	**NPV**	**PPV**	**NPV**
5%	64·4%	99·8%	56·6%	99·6%
10%	79·2%	99·6%	73·3%	99·1%
20%	89·6%	99·0%	86·1%	97·9%
30%	93·6%	98·3%	91·4%	96·4%
40%	95·8%	97·4%	94·3%	94·6%
50%	97·2%	96·1%	96·1%	92·1%
60%	98·1%	94·3%	97·4%	88·6%
70%	98·8%	91·4%	98·3%	83·3%
80%	99·3%	86·2%	99·0%	74·4%
90%	99·7%	73·5%	99·6%	56·3%

Note. PPV, positive predictive value; NPV, negative predictive value. Predictive values are calculated using sensitivity of 96·1% and specificity of 97·2% in symptomatic males, and 91·7% sensitivity and 96·3% specificity in symptomatic females as observed in this study.

Table S2. NG-MAST sequence types detected by NG LFA (n=168)

Sequence type	Number (%)
689	3
2187	2
2670	2
2975	2
6216	1
10107	1
12578	1
19216	3
20367	2
21045	1
21046	1
21047	2
21048	1
21052	1
21053	3
21054	1
21055	1
21056	1
21057	1
21058	1
21059	1
21060	1
21061	1
21062	4

21186	1
21187	2
21188	1
21190	1
21191	1
21192	2
21193	1
21194	1
21195	4
21198	1
21199	1
21200	1
21202	1
21203	1
21204	1
21205	1
21206	1
21207	1
21208	1
21209	1
21210	1
21211	3
21212	2
21245	1
21246	1

21247	1
21248	1
21250	1
21251	1
21252	1
21253	23
21254	6
21256	1
21257	1
21258	1
21259	1
21261	1
21262	1
21263	1
21264	1
21265	2
21266	1
21267	1
21268	1
21269	1
21270	1
21271	1
21272	1
21273	2
21274	1

21275	1
21277	2
21278	1
21279	1
21306	1
21307	2
21309	2
21312	2
21313	3
21314	1
21315	1
21316	1
21317	1
21318	1
21320	1
21321	2
21322	1
21324	1
21325	1
21326	2
TBA	1
Mixed NG-MAST types	1
Typing failed	6
No *Neisseria gonorrhoeae* DNA	7
**Total**	**168**
